# Efficacy of internet-based peer education for postpartum depression: study protocol of a randomized controlled trial

**DOI:** 10.3389/fpubh.2025.1630303

**Published:** 2025-09-25

**Authors:** Yu-Mei Zhou, Xiao-Wan Xiong, Han Zhou, Xiao-Ming Ma, Bing Yan, Jiang-Ning Qiu, Xiao-Yi Chen, Liu-Juan Zhang, Yan-Fang Wei, Xiao-Bing Zheng, Lin-Qing Li, Shao-Jun Chen, Yin-Li Xu, Si-Yi Han, Yu-Fen Lin, Yu-Qin Xu, Yan-Hua Gou

**Affiliations:** Shenzhen Traditional Chinese Medicine Hospital, The Fourth Clinical Medical College of Guangzhou University of Chinese Medicine, Shenzhen, Guangdong, China

**Keywords:** internet-based, peer education, postpartum depression, randomized controlled trial, protocol

## Abstract

**Introduction:**

Postpartum depression (PPD) is widely and profoundly affecting family and social stability. But now the consultation rate of overall PPD population is still not ideal for practical reasons. This study observes the effectiveness of Internet-based peer education, a promising treatment approach that may effectively and fairly serve the PPD group, so as to benefit more people in the future.

**Objectives:**

This study integrates internet-based peer education to serve PPD groups effectively and equitably, aiming to benefit more people. It explores IPE’s efficacy vs. RHE in reducing symptom severity at 6-week intervention and 16-week follow-up.

**Methods:**

This study is a randomized, parallel, and controlled trial, consist of a 6-week treatment period along with a 12-week follow-up period. Eighty-eight patients diagnosed as PPD will be recruited. They will be randomly assigned to one of two groups, internet-based peer education (IPE) group and regular health education (RHE) group, in a 1:1 ratio. Both groups will receive RHE along the 6-week treatment, while subjects in IPE group will receive 6-week online peer education additionally. The primary outcome is the response rate based on scores of 17-item Hamilton Depression Rating Scale at the week 6 and 12. The secondary outcomes include Edinburgh Postpartum Depression Scale (EPDS), World Health Organization Quality of Life Scale Brief (WHOQOL-BREF) and Self-Efficacy to Manage Chronic Disease Scale (SEMCD). All these scales will be evaluated at baseline, 1, 2, 4, 6, and 12 weeks after the initiation of intervention.

**Conclusion:**

The results of this RCT on Internet-based peer education for postpartum depression will provide an assessment of the feasibility of the protocol and data for power calculations to inform the development of a larger scale trial.

## 1 Introduction

As known, pregnancy is a complicated and vulnerable period for women, and it comes with a lot of unexpected difficulties (1). Postpartum depression (PPD) is one of the most common complications in perinatal period (2), classified as a subtype of major depressive disorder (MDD) with postpartum onset in American Psychiatric Association’s Diagnostic and Statistical Manual of Mental Disorders, fourth edition (DSM-IV) (3).

In high-income countries, the prevalence of postpartum depression (PPD) ranges from 6.9 to 12.9%, while in low- and middle-income countries, it exceeds 20% (4). This global variability underscores PPD as a pressing worldwide mental health concern, with far-reaching impacts on maternal health, child development, and family functioning (5, 6). Notably, prenatal depression has been identified as a key risk factor that significantly elevates the likelihood of PPD (7). Moreover, beyond its immediate effects on maternal wellbeing, PPD exhibits a striking comorbidity with depression in children and adolescents (8), further extending its public health relevance across the lifespan.

No matter in China or worldwide, women in postpartum period are increasingly vulnerable to suffer from poor mental health because of raising stress and less accessible social support (9). According to a population-based survey, after implementation of China’s universal two-child policy, it is pretty common that poor mental health outcomes still exist among women giving birth a second time (10). Unfortunately, due to a variety of reasons, such as lack of knowledge (11), perceived stigma (12), and health care system barriers (13), mothers with PPD prefer not to seek professional help.

What is inspiring, peer education services greatly benefit for recovery of people with mental health problems (6). Peer education is a structured intervention where individuals with lived experience of a specific condition (or similar life circumstances) provide support, share practical skills, and facilitate empowerment among those currently facing similar challenges (14). Peer educators can act as positive role for whom are currently experiencing mental distress by sharing real and useful skills they have acquired during their own experiences (9). It has to be notified that peer educators could not provide mental counsel but the information about depression, treatments, self-management and motivation for them to seek for needed professional services (10). But in other hand, peer educators may be more effective than mental health professionals at reducing stigma, enhancing attitudes toward mental health therapy because they share demographics with consumers (e.g., gender, age, income, etc.) and have personally experience involving mental illness (11). Moreover, peer education can help vulnerable groups that require mental health care but may feel excluded from the mainstream mental health system (12).

In this Internet age, a huge number of people can be offered low-threshold therapies online, regardless of time or location (15). A study shows that psychiatric patients use the Internet as frequently as the general population for engaging in online programs and accessing online health-related information (16). In addition, human support has repeatedly been shown to have a positive effect on effectiveness of and adherence to Internet-based interventions (17, 18). It has been shown in mental disorders that support provided by clinicians or coaches via telephone, email, and chat rooms could enhance adherence (19, 20). Consequently, online supportive program might be a promising approach to improve the accessibility, fairness, completeness, and quality of treatment for PPD population.

In the contemporary digital age, the relationship between peer education and internet—based interventions is intricate and mutually beneficial. Internet—based interventions offer a novel and far—reaching platform for the implementation and expansion of peer education.

Through online forums, instant messaging, and pre—recorded videos, they can disseminate information, share their personal experiences, and provide continuous motivation to those in need, regardless of physical location.

So the integration of internet services into mental health care has shown notable developments, particularly in leveraging digital platforms to address various psychological issues. Studies have explored the impact of internet use on mental health, with research on older adults indicating that internet engagement can influence psychological wellbeing through mechanisms like social equity perception (21), while also highlighting evolving research trends in this domain (22).

In healthcare services, internet-based peer education has emerged as a promising intervention. For instance, continuous peer education via online platforms has been applied to improve negative emotions, sleep quality, and self-care abilities among abortion patient (23). Similarly, “internet +” peer education models have effectively enhanced self-management behaviors and disease control in type 2 diabetes patients (24). These cases demonstrate the potential of digital peer support in complementing traditional healthcare.

However, a significant knowledge gap exists regarding the application of internet-based peer education in treating postpartum depression. While existing literature covers other populations and health issues, there is a lack of in-depth research on how such digital peer interventions can be tailored to address postpartum depression specifically within the Chinese context, leaving a critical area unexplored for optimizing mental health support in this vulnerable group.

This study aims to combine peer education with Internet-based intervention, maximize the advantages of two treatments, and explore a treatment approach that can effectively and fairly serve the PPD group, so as to benefit a wider range of population in the future.

The primary objective of the study is to investigate the efficacy of the Internet-based Peer Education (IPE) regarding changes in symptom severity compared to control group using Regular Health Education (RHE) during a 6 weeks intervention period and at the follow-up assessments at 12 weeks. Referring to previous evidence, we hypothesize that the IPE group will be superior compared to RHE group in reducing depressive symptoms directly after the 6 weeks of intervention, and at follow-up.

The secondary objective is to examine health related quality of life, the self-management ability and safety. We hypothesize, that the IPE group will lead to a significantly greater quality of life, better self-management and less adverse event than RHE group.

## 2 Methods

### 2.1 Study design

**Figure 1:**
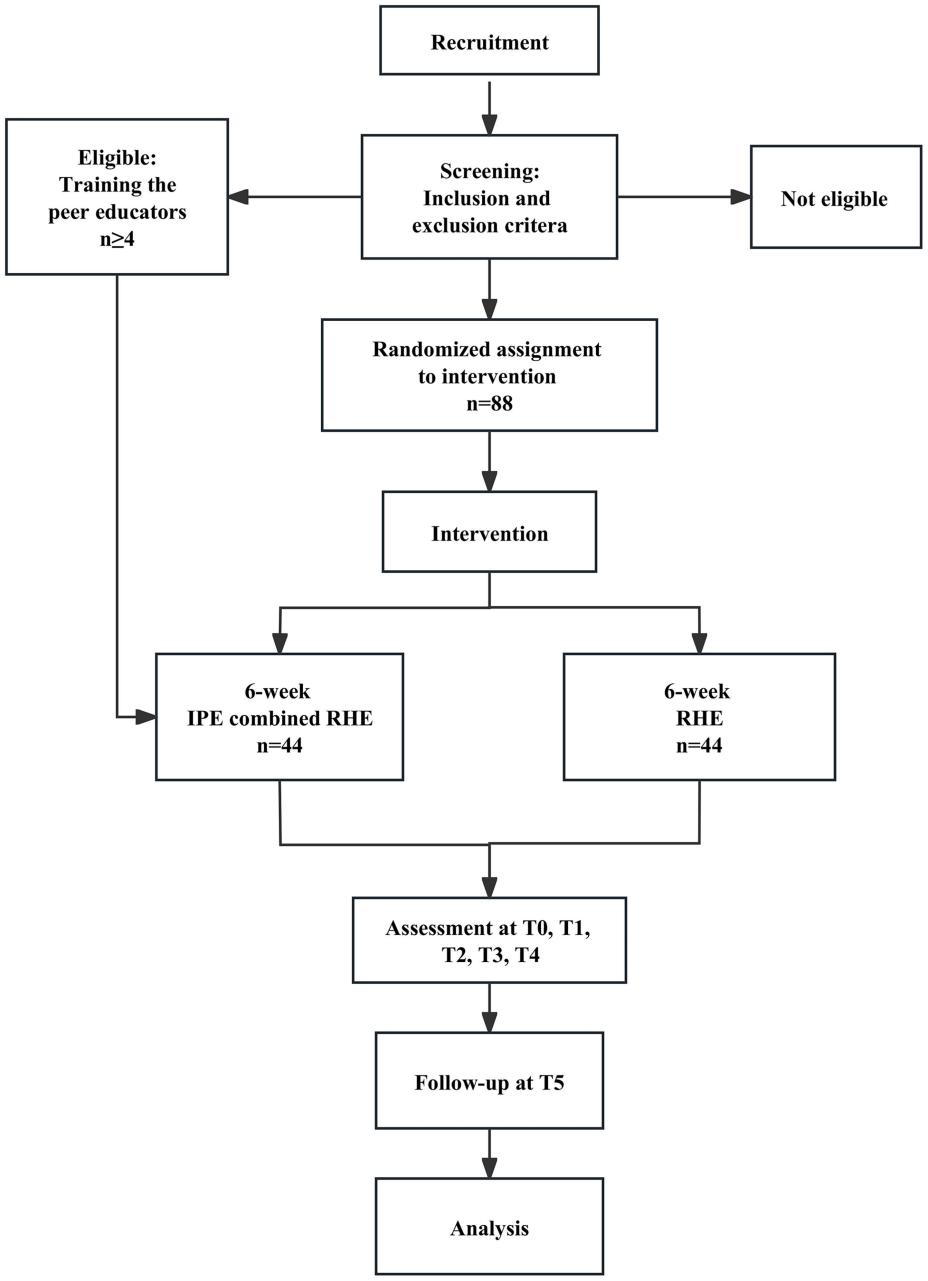
Trial flow.

This is a two-arm randomized controlled clinical trial (RCT) of parallel design comparing the effect and safety of a guided Internet-based peer education (IPE) with regular health education (RHE) after the 6-week intervention and at 12-week follow-up (Figure 1). All participants will receive RHE. Participants of the intervention group will additionally receive the IPE. Those evaluated tools containing 17-item Hamilton Depression Rating Scale (HDRS17), Edinburgh Postnatal Depression Scale (EPDS) (25), WHOQOL, Self-Efficacy for Managing Chronic Disease Scale (SMCDS).

This clinical trial is conducted and reported in accordance with the CONSORT-supplement for pragmatic RCTs, and is carried out in Shenzhen Traditional Chinese Medicine Hospital, a tertiary hospital, in July, 2023 to March, 2025. The trial has been approved by the institutional review board of the Shenzhen Traditional Chinese Medicine Hospital Ethics Committee (No. K2023-029-01). This study has been registered on June 19, 2023, with the Chinese Clinical Trial Registry, identification number ChiCTR2300072601.

Recruitment has begun at July, 2023 and will continue until the target sample size has been reached before March 2025. Different recruitment channels will be used: The study will be promoted primarily by WeChat Official Account of Shenzhen Traditional Chinese Medicine Hospital to all subscribers. And persons with interests will finish an online-pre-screening questionnaire then those who meet the criteria will be contacted for further investigation. Besides, participants will be recruited from the Postpartum Pelvic Floor Rehabilitation Clinic. After primary screening with EPDS, those score over 12 will be given the leaflets of this subject by their attending doctors. With this recruitment strategy, we will recruit a sample consisting of patients searching either online help or seeking help from clinic.

### 2.2 Inclusion and exclusion criteria

#### 2.2.1 Inclusion criteria

Patients who provide written consent will be included in the study if they meet the following criteria: (1) age 20–49; (2) meet the Diagnostic and Statistical Manual of Mental Disorders (DSM-IV) criteria for postpartum depression, diagnosed as PPD by a psychiatrist; (3) score of EPDS >12 points, score of HDRS17 > 7, ≤24; (4) volunteer to participate in this study, and can complete the scale evaluation; (5) the onset occurs within 12 months after childbirth.

#### 2.2.2 Exclusion criteria

Patients will be excluded who meet criteria for (1) bipolar disorder (according to DSM-IV diagnostic criteria), severe mental disorders such as schizophrenia; (2) with intellectual disability or difficulty in understanding the content of the questionnaire due to brain disease or other reasons, or unable to effectively talk; (3) pregnancy; (4) HDRS-17 suicidality item score >2; (5) suicidal behavior in the past year.

### 2.3 Consent procedure

Whose EPDS score over 12 will be introduced to a psychiatrist for further diagnosis of PPD. After diagnosis, researchers will preliminarily introduce this program through the mobile phone, then invite the volunteer for a face-to-face interview. All interviews will be launched in a quiet and dedicated room.

First, the researcher explained the program in detail to the interviewee in a one-to-one setting and determined if the interviewee met all the inclusion criteria and did not meet the exclusion criteria. If all conditions were met, the researcher would use the 17-item Hamilton Depression Rating Scale (HDRS17) to assess depressive symptoms and ask the interviewee whether she would be willing to participate. After clear understanding the benefits and potential risks of this research, those who are willing to participate in will sign written informed consent. Participants will be not compensated for study participation.

### 2.4 Randomization and allocation

The randomization was conducted using a computer-generated random number table (produced by SPSS 26.0 software) to ensure the unpredictability of group assignments. A total of 88 random numbers were generated, and each number was correspondingly assigned to either the intervention group (Internet-based peer education group) or the control group in a 1:1 ratio, with 44 subjects allocated to each group.

For allocation concealment, the research assistant who was not involved in subject recruitment prepared sequentially numbered opaque brown paper envelopes. Each envelope contained a card indicating the subject number, the generated random number, and the corresponding group assignment. These envelopes were sealed and stored in a locked cabinet to prevent premature disclosure.

When a qualified subject was enrolled, the researcher opened the envelopes in strict sequence according to the enrollment order (i.e., the first enrolled subject received the first envelope, the second subject the second envelope, and so on). The subject was then allocated to the group specified in the opened envelope. This procedure ensured that neither the researchers nor the subjects could predict the group assignment before enrollment, thus minimizing selection bias.

### 2.5 Blinding

This study obeys the principle of blinding, and the separation of researchers, therapists and statisticians will be strictly implemented throughout the study. The efficacy evaluation during the observation is conducted by dedicated raters (research doctors, nurses, or postgraduates) who are unaware of the grouping status. For statistician blinding, the statisticians involved in data analysis are also kept blind to the grouping information. In case of emergency, the researcher could inform the doctor of the subject’s condition, so that the appropriate treatment could be taken. An emergency envelope with the subject’s random number and treatment plan will be prepared in advance, so that the doctor could open it in necessary.

### 2.6 Interventions

#### 2.6.1 Internet-based peer education

On the basis of RHE, Internet-based peer education will be used for therapeutic intervention. Participants in treatment group will receive RHE and IPE. The specific methods of IPE are as follows:

##### 2.6.1.1 Intervention materials

(1) Digital platforms: WeChat (a widely used social communication application), utilized for creating dedicated education groups and facilitating real-time interactions.
(2) Educational content: Articles and videos covering postpartum health (diet, exercise, neonatal feeding skills), mental health knowledge, emotional management techniques, and peer self-management experiences.
(3) Training materials: Curricula for peer educator training, including modules on postpartum health, mental health, and emotional management, developed by the steering group.

##### 2.6.1.2 Procedures

(1) Steering group establishment: A multidisciplinary team is formed to oversee intervention design, peer educator training, and quality assurance.
(2) Peer educator selection and training: Eligible PPD patients are recruited, trained over 2 weeks, and assessed for competence before participation.
(3) Group formation: Enrolled subjects are assigned to WeChat education groups (maximum 11 subjects per group) containing steering group members, peer educators, and participants.
(4) Intervention implementation: Under the leadership of the guidance team, peer educators are trained and supervised to implement interventions for the subjects. The intervention measures include:
i. The guidance team disseminates curated postpartum dietary, exercise, and psychological adjustment content every Monday, Wednesday, and Friday, with a monthly communication plan;
ii. Peer educators provide weekly updates on member dynamics and needs every Friday, with the guidance team responding and adjusting within two working days;
iii. A 60–90 min group video chat is organized every Sunday evening, following an interactive process of “sharing—Q&A—summary”;
vi. Peer educators focus on experience sharing, presenting emotional management case studies every Tuesday and Thursday, and conducting monthly thematic sessions (e.g., “Postpartum Sleep and Emotional Regulation”), fostering a supportive atmosphere through positive reinforcement to encourage subject participation and mutual assistance.
(5) Safety protocol: Intervention is terminated immediately if participants exhibit suicidal tendencies or harmful behaviors, with referral to psychiatric care.

##### 2.6.1.3 Provider credentials

(1) Steering group: Comprises physicians with attending physician or higher professional titles (with extensive clinical experience) and registered nurses with a bachelor’s degree and ≥5 years of work experience (proficient in communication). Psychotherapists are also included to support training and content development.
(2) Peer educators: PPD patients meeting the following criteria: (i) cheerful personality with strong communication and organizational skills; (ii) high motivation and sense of responsibility; (iii) high school education or above, proficient in using smartphones; (iv) demonstrated effective postpartum depression self-management. They must pass a post-training assessment on health knowledge to qualify.

##### 2.6.1.4 Methods of intervention delivery

(1) All interventions are delivered online via WeChat, including;
(2) Asynchronous content sharing (articles, videos) in designated groups Synchronous video group chats (weekly) for interactive discussions.
(3) Bidirectional feedback: Peer educators regularly report to the steering group on group dynamics and content effectiveness, enabling real-time adjustments.

##### 2.6.1.5 Intervention schedule

(1) Training phase: 2 weeks, with 3 sessions/week (2 h/session) for peer educator training (group training and individual counseling).
(2) Intervention phase: 6 weeks, with: Weekly sharing of educational articles/videos by peer educators.
(3) One weekly video group chat (duration not specified, but structured to address self-management issues and experience sharing).

#### 2.6.2 Competency assessment of peer educators

The competency assessment of peer educators is primarily conducted across six dimensions: knowledge proficiency, communication and guidance skills, organizational and coordination capabilities, problem-solving abilities, sense of responsibility and execution, and professional competence. Detailed evaluation criteria are specified in the Supplementary materials **“**Competency Assessment of Peer Educators.” A 100-point scoring system is employed to quantify peer educators’ competencies, with performance indicators weighted as follows: knowledge proficiency accounts for 30%, communication and guidance skills for 25%, organizational and coordination capabilities for 15%, problem-solving abilities for 15%, sense of responsibility and execution for 10%, and professional competence for 5%. A total score of 80 points or above indicates competency qualification for peer educator responsibilities; scores between 60 and 79 points signify the need for targeted retraining; scores below 60 points denote disqualification from peer educator roles.

#### 2.6.3 Process evaluation plan

##### 2.6.3.1 Qualitative tools

###### 2.6.3.1.1 Outline of semi-structured interview (for peer educators)

(1) Basic information: including the duration of peer education work, the specific time of participating in this project, etc.
(2) Interaction Effects: “What were the three most effective moments in your group interaction this week? Describe the situation and why in detail.” “What is the biggest communication barrier you have faced when guiding group members to communicate? What coping strategies did you try? How did it work?”
(3) Personal feelings: “In the process of peer education, what links make you feel a sense of achievement? What aspects of peer education make you feel stressed?” “Do you think your current abilities are well suited to the role of peer educator? If so, what are the main areas of weakness?”

###### 2.6.3.1.2 Focus group discussion guide (for subjects)

(1) Activity format Acceptance: “How satisfied are you overall with the current activity format within the group? Explain why.” “What forms of activity do you wish to add or adjust? Why?”
(2) Feeling of Content: “What content does the group share that makes you feel understood and supported? Give an example.” “Is there any content that makes you feel offended or uncomfortable? What was it, specifically? Why do you feel this way?”
(3) Engage in motivation: “What makes you want to engage in group interactions and activities?” “What factors decrease your motivation to participate?”

###### 2.6.3.1.3 Guide template for electronic reflection diary (for both parties)

Daily Diary: “What was the one thing that impressed you most about your in-group communication today? How did it make you feel?” “Did you take the initiative to speak or participate in the group today? If not, what is the reason?”

Weekly summary: “What have you learned or learned from your learning and interaction in the group this week?” “How did you feel about the interaction in the group this week? What can you improve on?”

###### 2.6.3.1.4 Anonymous feedback box (online)

Set up a simple feedback entrance, suggesting the direction of the content of feedback, such as “I have thoughts on the speech of a member of the group,” “I feel that a certain activity is unreasonable,” “I have any other suggestions or complaints about the interaction in the group,” etc., and the feedback form is not limited, such as text and voice.

##### 2.6.3.2 Time node of data collection

(1) First assessment: 1 week after the intervention, participants had initially adapted to the group environment and interaction mode, and could provide some valuable feedback, mainly to understand the initial acceptability and participation.
(2) Stage evaluation: the intervention was carried out at the third and sixth week, and the changes of acceptability, participation and negative dynamics were tracked through multiple assessments in the process of intervention, so as to find and adjust problems in time.
(3) Summative evaluation: after the intervention, the feedback of the whole intervention process was collected comprehensively, the effectiveness, existing problems and improvements of the intervention were summarized, and the experience reference for subsequent related interventions was provided.
(4) Collect in real time: an electronic reflection diary encourages both parties to submit at a fixed time daily or weekly; Anonymous feedback boxes support participants to submit feedback at any time, ensuring that emergent feelings and questions are captured in a timely manner.

##### 2.6.3.3 Data analysis methods

(1) Data collation: arrange special personnel to transcribe semi-structured interview recordings and focus group discussion recordings into text data in time, classify and sort out the contents of electronic reflection diary and anonymous feedback box to remove duplicate or invalid information and ensure the completeness and accuracy of data.
(2) Coding and theme extraction: Thematic analysis was used by two independent psychotherapists. First of all, they read all the materials and were familiar with the content. After open coding, meaningful sentences and paragraphs were marked with different concepts. Then axis coding was performed to classify related concepts into sub-themes. Finally, selection coding was carried out to extract three core themes of “acceptability,” “participation” and “negative dynamics” and their specific subthemes.
(3) Consistency test: after the two psychotherapists finished coding, the coding consistency coefficient was calculated. If the coefficient was lower than 0.8, the differences were discussed together, and the final coding result was determined after reaching a consensus to ensure the reliability of the analysis.
(4) Results presentation and application: According to the coding results, the process evaluation brief was formed, and the specific performance and occurrence frequency of each theme were elaborated in detail. The brief was presented to the steering group, which adapted the intervention based on the intervention objectives and the actual situation. For example, if there is a lot of feedback in the “acceptability” theme about “the weekly theme task challenge is too difficult,” reduce the difficulty of the task; If “anxiety caused by the comparison of parenting experience among members” appeared frequently in the “negative dynamic,” the relevant activity links would be adjusted.

#### 2.6.4 Quality control

##### 2.6.4.1 Double-track system of content review

A strict double-track system of content review is established. All articles, videos and other materials to be shared in the group must be reviewed by both professional doctors and psychotherapists in the steering group. Focus on screening inappropriate expressions that may cause maternal anxiety, such as “you must adhere to exclusive breastfeeding, or you are an incompetent mother” and “you should quickly recover your figure after childbirth, or you will be rejected.”

##### 2.6.4.2 Dynamic evaluation system

Build a comprehensive dynamic evaluation system, and use a simple and intuitive “emotional thermometer” scale (0–10 points) to quickly evaluate the members’ emotions every week. If the member’s score is ≥7, the peer educator should immediately conduct a 15-min private chat with the member to deeply understand the causes of his or her emotional fluctuations, and provide personalized psychological support and suggestions. The emotional trend of the group was summarized and analyzed every month, and a detailed emotional trend map was drawn. Based on the trend chart, the steering group was keen to judge whether the focus of education content needed to be adjusted. For example, if the score of “sleep disturbance” dimension was high for two consecutive weeks, the thematic content on postpartum sleep improvement should be added in time, including sleep environment construction and sharing of sleep AIDS.

##### 2.6.4.3 Withdrawal management plan

In view of the possible loss of members, the “step-by-step retention” process is carefully designed. When a member fails to participate in the group activity for the first time, the peer educator sends a private letter in time, asks the reason in a friendly way, and expresses concern and encouragement; If the members were absent from the group activities for two consecutive times, the members of the steering group would conduct telephone interviews in person to understand the real thoughts and difficulties of the members, and try their best to provide help. If the member is confirmed to withdraw, the “personalized resource package” will be sent to the member according to their needs during the participation period, including targeted psychological adjustment materials, community support resources information, etc.

#### 2.6.5 Regular health education

The regular health education (RHE) in the study is further elaborated as follows:

(1) Providers: It is delivered by uniformly trained medical staff, who possess professional knowledge in obstetrics, gynecology, and mental health to ensure the accuracy and effectiveness of the education.
(2) Implementation Methods: The education is mainly conducted through telephone interviews and regular outpatient interviews. The frequency of telephone interviews is set at once every 3 days in the first 2 weeks, and then once a week for the remaining 4 weeks, each lasting 15–20 min. Regular outpatient interviews are scheduled once every 2 weeks, each lasting 30–40 min, allowing for more in-depth communication and assessment.
(3) Specific Content: Postpartum health education includes guidance on postpartum recovery exercises, dietary nutrition (e.g., recommended foods for lactation and avoiding irritating foods), personal hygiene care, and management of common postpartum physical discomforts (e.g., abdominal pain, breast swelling). Mental health knowledge covers the causes, symptoms, and self-assessment methods of postpartum depression, as well as basic emotion regulation techniques (e.g., deep breathing, progressive muscle relaxation). Social support education involves helping participants identify available social support resources (e.g., family, friends, community support groups) and teaching them how to effectively seek and utilize these supports. Family member education aims to guide family members (especially spouses and parents) to recognize the importance of their role in the postpartum period, learn to provide emotional care and practical assistance to the parturient, and create a harmonious family environment conducive to her recovery.
(4) Duration: The RHE intervention, same as IPE, lasts for 6 weeks to ensure continuous support for the parturient’s postpartum recovery.

#### 2.6.6 Alternative therapeutic approaches

During the study period, participants are not prohibited from receiving treatments other than the specified intervention. In cases of aggravated symptoms requiring antidepressant therapy, participants will be referred to psychiatric hospitals, where antidepressants will be prescribed by specialist psychiatrists. All details of antidepressant use or other treatments (including type, dosage, administration frequency, duration, and initiation reasons) will be documented in the CRF. These data will be incorporated into the statistical analysis to account for potential confounding from concurrent treatments.

### 2.7 Quality control

The study is designed in accordance with the declaration of Helsinki (26). Researchers are divided by functions into recruitment, assessment, and treatment. All researchers will be trained before participating in this trial to ensure the consistency of assessment and treatment measures.

Researchers in charge of recruitment are a psychologist and a gynecologist, and they will develop standard operating procedures (SOPs) for better retention of subjects according to the recruiting situation.

Assessment researchers will be trained by psychiatrists on how to correctly use the HDRS17. Assessments at all-time points of one subject will be performed by the same researcher. Phone calls will be carried out not only for HDRS17 assessment, but also to improve compliance with the interventions and online self-report questionnaires. To keep the study procedures consistent across different researchers, SOPs will be developed and all researchers will be trained on the SOPs. All information will be recorded completely, standardly and in a timely manner on the CRF. We will randomly carry out self-inspection of CRFs to ensure completeness, accuracy and timeliness. Two researchers will transfer the data into a database and then cleaned and prepared the database.

Before onset of the trial, all selected steering group members will be organized to meet together, psychological expert will be invited to guide the content of mental health education, the management of the subjects, and the measures of emergency situations, so as to formulate management rules. After the start of the study, the steering group members should summarize the implementation of IPE in the steering WeChat group weekly, and revise the management rules if necessary to ensure the quality of IPE implementation.

### 2.8 Assessment

Assessments take place before the onset of the intervention period (T0), 1 week (T1), 2 weeks (T2), 4 weeks (T3) after the intervention has started, and at the end of the intervention (6 weeks after start of the intervention; T4). Follow-up will take place at 12 weeks after the end of the intervention (T5) (Table 1). Each assessment consists of an online questionnaire and a telephone interview. Except HDRS17, the EPDS, WHOQOL and SMCDS are self-report scales, which will be filled out online at T1-T5. The access of online questionnaires will be sent to subjects by researchers in charge of assessment, at the assessment nodes after inclusion. HDRS17 will be assessed by researchers through telephone interview with subjects.

**Table 1:** Overview of assessment points and measurements used.

	Online screening	Face-to-face interview	T0	T1	T2	T3	T4	T5
			-7 ~ 0d	1w ± 3d	2w ± 3d	4w ± 3d	6w ± 3d	12w ± 3d
In−/exclusion criteria	×							
Witten consent form		×						
Sociodemographic information		×						
Regular health education			×	×	×	×	×	
Internet-based peer education			×	×	×	×	×	
Edinburgh Postpartum Depression Scale			×	×	×	×	×	×
17-item Hamilton Depression Rating Scale			×	×	×	×	×	×
World Health Organization Quality of Life Scale Brief			×	×	×	×	×	×
Self-Efficacy to Manage Chronic Disease Scale			×	×	×	×	×	×
Medical history, medication, therapies		×	×	×	×	×	×	×
Adverse event		×	×	×	×	×	×	×
Assessment of adherence		×	×	×	×	×	×	×
Completion assessment		×	×	×	×	×	×	×
Review by investigator		×	×	×	×	×	×	×

### 2.9 Sample size

The sample size has been calculated according to the results from a previous clinical trial (27). The primary outcome in present study is the response rate at the end of 6-week intervention. Referring to the study of Uebelacker et al. (27), the response rate of health education group is 28.3% (responders). This study assumed that the effective rate of IPE combined with RHE was 70%. A two-sided test is required (25), *α* = 0.05, the sample size ratio of the two groups N1: N2 = 1:1 (i.e., the number of cases in the two groups is equal), *β* = 0.2, and the power of the test is 1-*β* = 80%. The analysis result is N1 = N2 = 38 cases, and 44 cases are included in each group according to the dropout rate of 15%. Therefore, a total of 44 patients/group ×2 groups = 88 patients will be included in this study.

### 2.10 Statistical analysis plan

The statistical analysis will be conducted by independent statisticians who will be blinded to group allocation and intervention methods. Before data analysis, the research group will draw up a statistical plan, including the required data and method of data processing. For Categorical data, we performed the Chi-square (x^2^) test for between-group differences. For continuous and normally distributed data, we performed Student’s *t*-test for between-group differences and paired t test for within-group differences. If data were distributed nonnormally, the Mann–Whitney U test for between-group differences and the Wilcoxon test for within-group differences would be used. The normally distributed data were expressed as the mean ± standard deviation. Nonnormally distributed data would be expressed as the median or interquartile range (IQR). For categorical variables, the X^2^ test will be used to examine the between-group differences, and percentages and frequencies will be presented to describe the effect size.

For longitudinal data analysis considering the repeated measurements at assessment nodes in this study, the Generalized Estimating Equations (GEE) model will be employed.

Fixed effects structure: It will include the group variable (experimental vs. control), time points (six assessment nodes as a categorical variable), and the interaction term between group and time points. Additionally, potential confounding variables (e.g., baseline characteristics that show imbalance between groups) will be incorporated into the model as covariates to adjust for their effects.

Correlation structure: Given the repeated measurements within individuals over time, the exchangeable (compound symmetry) correlation structure will be initially assumed, which posits that the correlation between any two measurements from the same individual is equal. Sensitivity analyses will be conducted using other correlation structures (e.g., autoregressive of order one, which assumes a higher correlation between measurements that are closer in time) to assess the robustness of the results. The working correlation matrix will be estimated based on the observed data during model fitting.

All clinical data analyses will be performed using SPSS V.22.0 software. Missing data will be handled using the multiple imputation method. A two-tailed test will be conducted, and a *p* < 0.05 will be considered statistically significant.

### 2.11 Primary outcome

The response rate based on 17-item Hamilton Depression Rating Scale (HDRS17) score at week 6 is considered primary outcomes. Response rate is defined as the percentage resulting from the dividing of the scores at baseline from the difference between those of baseline and week 6. The response rate reaches or over 50%, the patient will be defined as a responder; otherwise, the other patients will be considered non-responders. The formula for calculating the response rate is as follows:


$$\text{Response Rate}=\frac{\left(\text{HDRS}17\;\text{score}\;\mathrm{at}\;8\mathrm{th}\;\text{week}-\text{HDRS}17\;\text{score}\;\mathrm{at}\;\text{baseline}\right)}{\text{HDRS}17\;\text{score}\;\mathrm{at}\;\text{baseline}}\times 100\%$$

### 2.12 Secondary outcome

(1) Edinburgh Postpartum Depression Scale (EPDS) (25). It is considered the most frequently validated instrument to screen for perinatal depression. The scale consists of 10 items, mainly concerning the factors like depression, anxiety, anhedonia and self-harm/suicide. Higher scores indicate more severe symptoms of postpartum depression.
(2) World Health Organization Quality of Life Scale Brief (WHOQOL-BREF) (28). This standardized tool conceptualizes quality of life as personal perception. Physical health, psychological health, social interactions, and environment are the four dimensions that make up WHOQOL-BREF. There are three to eight items in per domain. Additionally, two generic questions on health satisfaction and overall quality of life (Q1 and Q2) are provided. With the exception of 3 items—pain and discomfort, the need for treatment, and negative feelings—each item is based on self-report and scored on a scale of one to five, with higher scores indicating higher quality of life.
(3) Self-Efficacy to Manage Chronic Disease Scale (SEMCD) (29). This patient reported scale mainly evaluate symptoms of chronic conditions, functioning, and self-management. This self-efficacy scale for chronic disease patients was developed by Stanford University in the United States. The scale contains 6 items, each item ranging from 1 to 10, with 1 indicating no confidence at all and 10 indicating great confidence. The average score of the 6 items reflected the average level of self-efficacy of patients. A score of 1–3 indicates a low level of self-efficacy, a score of 4–7 indicates a medium level of self-efficacy, and a score of 8–10 indicates a high level of self-efficacy. Its Cronbach’sα coefficient is 0.91, with high reliability and wide application.

Scales information will be collected and managed by corresponding researchers, who are unaware of the group division. To exclude confounding factors, each subject will be evaluated by the same researcher in the duration of trial.

Evaluation time points: all scales will be evaluated at baseline, 1, 2, 4, 6, and 12 weeks after the initiation of intervention.

### 2.13 Safety

The steering group members and the assessment researchers will closely observe the psychological conditions of the peer educators and the participants during the whole course of trial. If an adverse event (AE) happened during intervention, it would be assessed and recorded in the case report form (CRF). The relevant adverse events may include deterioration of the depression, aggravating hopelessness, anxiety and somatic symptoms (et al., dizzy, pain). Serious adverse events include self-harm tendency, suicide attempt, and injury-infant behavior. Once a serious adverse event occurs, the participant will be immediately terminated from this trial and will be referred to a psychiatric clinic as soon as possible.

In addition to routine exclusion and monitoring criteria for suicidality, specific measures for symptom emergence during non-usual clinic hours are established. Participants will be provided with a 24/7 emergency hotline connected to on-call psychiatric professionals. Upon reporting suicidal symptoms, the on-call team will conduct immediate risk assessment via phone, dispatch emergency services if necessary, and coordinate with the participant’s primary caregiver for in-person supervision until professional intervention is available. Documentation of all interactions and follow-up actions will be maintained in the participant’s record.

### 2.14 Data management and monitoring

The information will be recorded on the CRFs accurately, quickly, and completely gathered. For confidentiality, patient information, like medical records, names, contact information, and ID numbers will be kept anonymous. All relevant materials will be archived for at least 5 years after publication and kept in settled place. Moreover, a data monitoring committee is made up of experienced professionals in the Shenzhen Traditional Chinese Medicine Hospital’s Good Clinical Practice Department. The committee takes charge in monitoring data collection, allocation concealment, and analyze trial progress on a regular basis. The modification or termination of the trial can be performed by the committee. The data monitoring committee is independent from the sponsor and has no conflict of interest.

### 2.15 Trial status

The recruitment of this trial has begun at July 2023 and will continue until the target sample size has been reached before March 2025. The version number and date of this protocol are v1.0, and March 20, 2023, respectively.

## 3 Discussion

The treatment of PPD should be of the high priority for public health systems due to its high prevalence and severe impact on a wide spectrum of family lives. However, limited to safety and accessibility of treatment, PPD is still undertreated (30).

Based on previous trials, the peer education has shown its promising efficacy of managing mental disorder and chronic diseases, such as coronary angiography with anxiety and depression (31), cancer patients with depression (32), and ankylosing spondylitis (33). In addition, several studies have shown that peer education has good acceptance among vulnerable groups, such as adolescents (34), the older adults (35), and postpartum women (36). Currently, guideline-recommended therapies for MDD, such as medication and psychological counseling, do not meet the needs of PPD patients. Since the amount of drugs in breast milk and the implications for infant health remains controversial, up to 50% of mothers refuse to take medications for their depressive symptoms (30). Additionally, psychological counseling is too expensive, unavailable, or inaccessible for women living in rural and remote area or low-income (37). Therefore, it is urgent to explore a safe, highly adherence, economical and easy accessed therapy for PPD population.

We integrate peer education and Internet-based intervention to make peer education more convenient, efficient and accessible for PPD group. In previous studies, Internet-based interventions have shown to be effective in the treatment of MDD (38, 39), especially mothers in third trimester (40) or of premature infants (41). Hence, the IPE for managing PPD is certainly feasible.

### 3.1 Limitation

Firstly, due to the characteristics of IPE intervention, it is not possible to blind patients, the efficacy may be affected by expectation of participants. Secondly, except the primary outcome, the self-reported scales are employed as secondary outcomes, so we will interpret this part of data conservatively.

### 3.2 Strengths

This study’s strengths include integrating internet-based intervention with peer education, enhancing accessibility for postpartum women. It targets a defined population (postpartum mothers within 1 year), addressing PPD vacant, filling knowledge on combined interventions for PPD.

## 4 Conclusion

In conclusion, this article presents a design for a RCT on Internet-based peer education for postpartum depression. The results of this trial will provide an assessment of the feasibility of the protocol and data for power calculations to inform the development of a larger scale trial.
