# Correction: Efficacy of internet-based peer education for postpartum depression: study protocol of a randomized controlled trial

**DOI:** 10.3389/fpubh.2025.1713708

**Published:** 2025-10-22

**Authors:** Yu-Mei Zhou, Xiao-Wan Xiong, Han Zhou, Xiao-Ming Ma, Bing Yan, Jiang-Ning Qiu, Xiao-Yi Chen, Liu-Juan Zhang, Yan-Fang Wei, Xiao-Bing Zheng, Lin-Qing Li, Shao-Jun Chen, Yin-Li Xu, Si-Yi Han, Yu-Fen Lin, Yu-Qin Xu, Yan-Hua Gou

**Affiliations:** Shenzhen Traditional Chinese Medicine Hospital, The Fourth Clinical Medical College of Guangzhou University of Chinese Medicine, Shenzhen, Guangdong, China

**Keywords:** internet-based, peer education, postpartum depression, randomized controlled trial, protocol

The Supplemental material file “Competency Assessment of Peer Educators” was omitted. The file has now been added to the original article.

The original version of this article has been updated.

