# Supplementary material for: Efficacy of internet-based peer education for postpartum depression: study protocol of a randomized controlled trial
Source: Front Public Health. 2025 Sep 25;13:1630303. doi: 10.3389/fpubh.2025.1630303 (PMC12507833; doi:10.3389/fpubh.2025.1630303)
Supplement: Supplementary file 1 [file Supplementary_file_1.docx]

**2.7.2 Competency Assessment of Peer Educators**

**2.7.2.1 Evaluation indicators**

(1) Mastery of knowledge: the mastery of postpartum depression related knowledge, including postpartum health, mental health and emotional management, was measured by knowledge questionnaire, and the mastery rate of core knowledge points was required to be no less than 90%.

(2) Communication and guidance skills: The ability to clearly and accurately convey information to peer group members, patiently answer members' questions, express in simple language, and adjust the communication method according to members' understanding. The timeliness (response rate within 24 hours is not less than 95%) and effectiveness (member satisfaction is not less than 85%) of responding to members' questions in the wechat group are important measures.

(3) Organization and coordination ability: ability to effectively organize online discussion, knowledge sharing and other activities of peer groups, reasonably arrange the time and content of activities, and mobilize the enthusiasm of members to participate in group activities. The participation rate of group activities should be no less than 70%.

(4) Problem-solving ability: in the face of contradictions and conflicts, negative emotions of members, knowledge understanding deviation and other problems in the group, can timely find and take appropriate ways to deal with them, and the success rate of problem solving is not less than 80%.

(5) Responsibility and execution: strictly abide by the responsibilities of peer educators, complete the learning of training content on time, submit reflection records regularly, remind members to review regularly, and the task completion rate should reach 100%.

(6) Professionalism: maintain a positive working attitude, respect each member of the group, protect the privacy of members, follow professional ethics, and do not violate ethical norms.

**2.7.2.2 Scoring methods**

(1) Knowledge mastery: based on the knowledge questionnaire evaluation score, the full score was 30 points, score = actual score of the questionnaire/total score of the questionnaire ×30.

(2) Communication and guidance ability: combined with the members' evaluation of the timeliness and effectiveness of the response, the full score was 25. Among them, the timeliness of response accounted for 10 points, and the score was calculated according to the response rate within 24 hours. The effectiveness of the response accounts for 15 points, and the score is calculated based on member satisfaction.

(3) Organization and coordination ability: according to the participation rate of group activities, the full score is 15 points, and the participation rate reaches 70%, 10 points are scored, 1 point is added for every 5% increase, the maximum is 15 points, and points are deducted for less than 70%.

(4) Problem handling ability: according to the success rate of problem solving, the full score is 15 points, the success rate reaches 80%, 10 points, 1 point is added for every 5% increase, the maximum is 15 points, and points are deducted proportionally for less than 80%.

(5) Responsibility and execution: Based on the task completion rate, the full score is 10 points, the task completion rate of 100% is 10 points, 1 point is deducted for every 5% decrease, and the minimum point is 0.

(6) Professional quality: by observing their performance in the work, the full score is 5 points, 5 points are given for no violation of ethical norms and positive attitude, points are deducted for minor problems, and 0 points is given for serious violations of ethical norms.

**2.7.2.3 Peer Educator process assessment Plan**

In order to ensure that peer educators continue to meet the ability requirements during the intervention period and ensure the compliance and quality stability of the intervention work, regular and irregular assessments were conducted during the intervention period.

(1) Regular assessment: peer educators were comprehensively evaluated using the performance indicators and scoring methods mentioned above in the second, fourth and sixth weeks of the intervention. Assessment by a professional team of doctors, college of nurse assessment team, by looking at WeChat group of member records, analysis, feedback, examination of peer educators reflection records, communicate with a peer educator methods such as collect relevant data, carried out in accordance with the criteria.

(2) Unscheduled evaluation: the evaluation team will randomly select time periods to evaluate the work performance of peer educators, focusing on the timeliness and effectiveness of their communication guidance, and the appropriateness of problem handling. If peer educators are found to have a significant decline in a performance indicator, remind and guide them in time, and focus on the improvement of this indicator in the follow-up regular evaluation.

(3) Data analysis: frequency analysis, central tendency analysis and dispersion analysis were carried out according to the scores of various performance indicators of peer educators' ability assessment. Through frequency analysis, the distribution of different score ranges is clearly presented, and the proportion of peer educators at different levels under each performance indicator is clarified. The mean, median and mode were used for central tendency analysis to accurately reflect the general level of the scores of various performance indicators. For example, the mean score of knowledge mastery can reflect the overall knowledge level of peer educators. Dispersion analysis using variance and standard deviation can measure the volatility of data, such as the standard deviation of communication and guidance ability scores, which can intuitively show the difference of peer educators in this ability. In addition, the statistical graphics, such as scale histogram, boxplot appears in the form of intuitive visual data characteristics, help the researchers quickly grasp the various performance indicators score distribution form and discrete. To ensure that the intervention work smoothly and stable quality.

(4) Processing: evaluation results for evaluating score in 60-79 of peer educators, set by the research team for its weak link personalized training plan, arrange for three days of intensive training and follow-up of individual coaching, training again after the evaluation, until qualified standard is reached. For two consecutive evaluation unqualified peer educators, cancel the qualification of peer educators, to select the right people from the peer group training and assessment.
